# Community Composition and Diversity of Intestinal Microbiota in Captive and Reintroduced Przewalski’s Horse (*Equus ferus przewalskii*)

**DOI:** 10.3389/fmicb.2019.01821

**Published:** 2019-08-07

**Authors:** Yimeng Li, Ke Zhang, Yang Liu, Kai Li, Defu Hu, Torsten Wronski

**Affiliations:** ^1^College of Nature Conservation, Beijing Forestry University, Beijing, China; ^2^School of Natural Sciences and Psychology, Liverpool John Moores University, Liverpool, United Kingdom

**Keywords:** bacterial community composition, diet quality, feeding regime, LEfSe analysis, symbiosis

## Abstract

Large and complex intestinal microbiota communities in hosts have profound effects on digestion and metabolism. To better understand the community structure of intestinal microbiota in Przewalski’s horse (*Equus ferus przewalskii*) under different feeding regimes, we compared bacterial diversity and composition between captive and reintroduced Przewalski’s horses, using high-throughput 16S-rRNA gene sequencing for identification. Reintroduced Przewalski’s horses were sampled in two Chinese nature reserves, i.e., Dunhuang Xihu Nature Reserve (DXNR; *n* = 8) in Gansu Province and Kalamaili Nature Reserve (KNR; *n* = 12) in Xinjiang Province, and compared to a captive population at the Przewalski’s Horse Breeding Center in Xinjiang (PHBC; *n* = 11). The composition of intestinal microbiota in Przewalski’s horses was significantly different at the three study sites. Observed species was lowest in DXNR, but highest in KNR. Lowest Shannon diversity was observed in DXNR, while in KNR and PHBC had a moderately high diversity; Simpson diversity showed an opposite trend compared with the Shannon index. Linear Discriminant Analysis effect size was used to determine differentially distributed bacterial taxa at each study site. The most dominant phyla of intestinal microbiota were similar in all feeding regimes, including mainly Firmicutes, Bacteroidetes, Verrucomicrobia, and Spirochaetes. Differing abundances of intestinal microbiota in Przewalski’s horses may be related to different food types at each study site, differences in diversity may be attributed to low quality food in DXNR. Results indicated that diet is one of the important factors that can influence the structure of intestinal microbiota communities in Przewalski’s horse. These findings combined with a detailed knowledge of the available and consumed food plant species could provide guidelines for the selection of potential future reintroduction sites.

## Introduction

The Przewalski’s horse (*Equus ferus przewalskii*; recently identified as a feral descendant of wild horses domesticated in today’s Kazakhstan, Gaunitz et al., 2018), was once considered extinct in China and Mongolia (Mohr, 1971; Bouman and Bouman, 1994). In 1945, only 31 horses survived in captivity but due to joint breeding efforts, their number had increased to over 1,500 individuals in the early 1990’s (Bouman and Bouman, 1994). In 2005, a first cooperative venture between European zoos and Mongolian scientists resulted in a successful reintroduction into their natural habitat in Mongolia, and as of 2014 there is an estimated free-ranging population of over 1,988 Przewalski’s horses (King et al., 2015). In China, reintroduction efforts started in 1985 with the establishment of the PHBC in Xinjiang. In 2001 the first reintroduction was realized with a release of 206 horses into the KNR in Xinjiang Province (Chen et al., 2008; Liu et al., 2014), followed by a second reintroduction of 40 horses into Dunhuang Xihu Nature Reserve (DXNR), Gansu Province, in 2010 (Wang et al., 2012; Liu et al., 2014). Concerns among conservationists that captive horses have altered morphological, behavioral, or genetic traits compromising fitness or changing the species’ functional role in their original habitat (O’Regan and Kitchner, 2005), were not verified (Ballou, 1994). At present, the most urgent conservation actions to be considered are the improvement of the population’s genetic diversity, to prevent losses due to stochastic events (i.e., severe winter), to prevent hybridization with domestic horses and to improve habitat quality (e.g., pasture and water; King et al., 2015). Pasture is one of the most important factors determining the survival of reintroduced Przewalski’s horses in the wild (Burnik Šturm et al., 2016; Kaczensky et al., 2017). Given that in captivity (i.e., prior to reintroduction), Przewalski’s horses were fed a standardized, but low variety diet, they encounter different habitats and a variety of different food types after release into the wild. This change of diet can cause adaptive responses of the intestinal microbiota (Metcalf et al., 2017). Until recently, nutritional research in Przewalski’s horse has mainly focused on dietary preferences, food selection, and feeding behavior of captive and reintroduced populations (Berger et al., 1999; Meng, 2007), but more recently also on dietary requirements prior to their extinction in the wild using stable isotopes (Burnik Šturm et al., 2016; Kaczensky et al., 2017). Lately, comparative studies on the dietary requirements of Przewalski’s horses extended their scope to the role of intestinal microbiota during digestion (Laho et al., 2013; Metcalf et al., 2017).

Intestinal microbiota have an important role in the hindgut of equines during digestion (Costa and Weese, 2012). The host provides a stable and nutritious environment to the microbiota, while at the same time relying on the capabilities of the microorganisms to break down structural carbohydrates such as cellulose, hemicellulose and pectin (Yeoman et al., 2011; Costa and Weese, 2012). Moreover, the intestinal microbiota is vital to the host’s fitness, including the proliferation of enterocytes, the protection against pathogens, and the production of secondary metabolites (Flint et al., 2008; Walter et al., 2011). The composition and abundance of intestinal microbiota varies considerably between individuals, depending on intrinsic factors such as age, physiological condition, life history and genetic set-up, but also on environmental factors such as food plants, dietary composition and other environmental parameters (e.g., season, climate, soil composition; Jandhyala et al., 2015). Environmental changes can sustainably modify the composition and metabolic activity of intestinal microbiota, and thus affect the host’s digestive ability and health (Conlon and Bird, 2014; Zhang and Yang, 2016). Amongst those intrinsic and environmental factors, diet is the most important aspect influencing intestinal microbiota in hindgut fermenters such as the Przewalski’s horse (Bäckhed et al., 2005; Turnbaugh et al., 2006). In recent years, a remarkable diversity of microbiota was discovered in the hindgut of equines, accumulating to about 750,000 high quality sequences that could be clustered into 5689 unique operational taxonomic units (OTUs; Dougal et al., 2014).

Research on intestinal microbiota is of great significance to understand the dietary fitness and to monitor the health status of host species. This applies particularly to species that were reintroduced into new environments after being kept in captivity for several generations. In this study, we focused on the intestinal microbiota of Przewalski’s horses in relation to their diet by comparing the diversity (richness and evenness) and community composition of intestinal microbiota between captivity (site PHBC) and two reintroduction sites (KNR and DXNR). Metcalf et al. (2017) compared the fecal microbiomes of Przewalski’s horses and domestic horses, showing that Przewalski’s horses have a more distinct and more diverse community of bacteria compared to that in domestic horses. This is likely due to higher plant variety and thus a more diversified diet at the reintroduction site of Przewalski’s horses, compared to the enclosure in which domestic horses were kept. Since food quality is higher, but dietary variety is lower in captivity, we predicted the intestinal microbiota richness and diversity to be lowest in the captive population of the PHBC, but higher at the two reintroduction sites in DXNR and KNR. Between reintroduction sites we expected microbiota richness and diversity to be higher in the KNR than in the DXNR, where climatic conditions (i.e., precipitation, evaporation) and soil composition (mineral content) entail lower nutrient quality, less diet variety and availability than in KNR (Meng, 2007; Chen et al., 2008; Wang et al., 2012). To determine differentially distributed bacterial taxa at each study site a Linear Discriminant Analysis (LDA) effect size (LEfSe) analysis was performed. Finally, non-metric multi-dimensional scaling (NMDS) and subsequent one-way analysis of similarity (ANOSIM) was used to identify similarities in OTU community composition between study sites. We expected significant differences in OTU composition, with larger variance between the PHBC population and the two reintroduced populations. Given that the food availability and variety (plant community structure), are higher in KNR (Meng, 2007; Chen et al., 2008; Wang et al., 2012), we expected the bacteria community to be more diverse than in the DXNR.

## Materials and Methods

### Ethics Statement

This study was carried out in accordance with the recommendations of the Institute of Animal Care and the Ethics Committee of Beijing Forestry University. The Ethics Committee of Beijing Forestry University also approved the protocol. The management authority of KNR, DXNR and the Xinjiang Przewalski’s Horse Breeding Center approved the collection of Przewalski’s horse fecal samples.

### Study Areas

Fecal samples were collected at two reintroduction sites: i.e., the KNR and the DXNR, as well as in the captivity of the PHBC. The KNR is located in northern Xinjiang Province (89°14′–89°36′E, 45°49′–46°4′N), with an average annual precipitation of 159.1 mm. Here, major food plants of Przewalski’s horses include the bunchgrass (*Stipa capillata*), Pamirian winterfat (*Ceratoides latens*), wormwood (*Artemisia* spp.) and *Anabasis brevifolia*, a salt-tolerant, woody xerophyte (Meng, 2007). The DXNR is located near Dunhuang City in western Gansu Province, (92°45′–93°50′E, 39°45′–40°36′N) with an average annual precipitation of 39.9 mm. Przewalski’s horses feed here on a relatively simple variety of plants, mainly reeds (*Phragmites australis*) and camelthorn (*Alhagi sparsifolia*) (Wang et al., 2012). The PHBC is located near Urumqi City in Xinjiang Province (88°45′–88°50′E, 44°10′–44°15′N), with an average annual precipitation of 160.2 mm. Food (alfalfa, mixed feed-concentrates) and drinking water were regularly supplied by the management. In winter, small amounts of carrot and corn flour were added (Ji, 2013). In July, the average temperature is 17–33°C and the average precipitation is 12 mm in DXNR. The temperature and precipitation are similar in KNR and PHBC, that is, the average temperature is 19–30°C and the average precipitation is 23 mm.

### Sample Collection

A total of 31 fresh fecal samples was collected in July 2018; twelve (6 males, 6 females) in KNR, eight (5 male, 3 female) in DXNR and eleven in the PHBC (5 males, 6 female). Sampled individuals were 4–8 years of age (for metadata see Supplementary Table S1). In PHBC only healthy individuals were sampled, ensuring that none of them was administered antibiotics or antiphlogistic drugs during the past 3 months. Sample collection at the two reintroduction sites was carried out from a vehicle whilst following different family groups. We observed the Przewalski’s horses at distances about 20–30 m waiting for a group member to defecate. After the group had moved on, fecal samples with similar consistency were collected into sterile centrifuge tubes, sealed, labeled and retained in a mobile refrigerator until taken to the laboratory for final storage at −80°C. DNA extraction was carried out within 1 week after sample collection.

### DNA Extraction, Purification and 16S-rRNA Gene Sequencing

Bacterial DNA was extracted using the QIAamp DNA Stool Mini Kit (QIAGEN, Hilden, Germany) according to the manufacturer’s protocol. The integrity of the nucleic acids was determined visually by electrophoresis on a 1.0% agarose gel containing ethidium bromide. The concentration and purity of each DNA extract were determined using a Qubit dsDNA HS Assay Kit (Life Technologies, Carlsbad, CA, United States). The extracted total DNA was preserved at −80°C. The V3-V4 region of the bacterial 16S-rRNA gene was amplified with the universal bacterial primers 341F (5′−*CCCTACACGACGCTCTTCCGATCTG*−3′) and 805R (5′−*GACTGGAGTTCCTTGGCACCCGAGAATTCCA*−3′; Jakobsson et al., 2014). PCR amplification was performed in a total volume of 50 μL, which contained 10 μL PCR buffer, 0.2 μL Q5 High-Fidelity DNA Polymerase, 10 μL High GC Enhancer, 1 μL dNTP, forward and reverse primers (1.5 μL each, 10 μM), 60 ng genome DNA and the remaining volume was ddH_2_O. Thermal cycling conditions were as follows: an initial denaturation at 95°C for 5 min, followed by 25 cycles of 95°C for 30 s, 50°C for 30 s, 72°C for 40 s, with a final extension at 72°C for 7 min. PCR products were mixed with the same volume of 2 × loading buffer and were subjected to a 1.8% agarose gel electrophoresis for detection. Samples with a bright main band of approximately 450 bp were chosen and mixed to reach equal ratios. Subsequently, the mixture of PCR products was purified using a GeneJET Gel Extraction Kit (Thermo Fisher Scientific, Waltham, MA, United States). Sequencing libraries were validated using an Agilent 2100 Bioanalyzer (Agilent Technologies, Palo Alto, CA, United States) and quantified with a Qubit 2.0 Fluorometer (Thermo Fisher Scientific). Finally, paired-end sequencing (2 × 250 bp) was conducted using an Illumina HiSeq 2500 platform (Illumina, Inc., San Diego, CA, United States) at Biomarker Technologies Corporation, Beijing, China.

Raw sequences were quality filtered under specific filtering conditions to obtain high-quality clean tags based on the QIIME (Version 1.8.0) quality control process. Sequences with less than 200 bp or that contained homopolymers longer than 8 bp were discarded. Chimera sequences were detected by comparing tags with the reference database [Ribosomal Database Project (RDP) Gold database] using the UCHIME (Version 4.2) and then removed. Only effective sequences were used in the final analysis. Sequences were grouped into OTUs using the clustering program UCLUST (Version 1.2.22) (Edgar, 2010) for *de novo* OTU picking and matched with the SILVA bacterial database (Quast et al., 2013) and pre-clustered at 97% sequence identity. Taxonomic classification into hierarchical groupings was obtained using the online RDP classifier with a confidence threshold of 80% (Wang et al., 2007). All raw sequences obtained during this study were submitted to the NCBI Sequence Read Archive (accession number SRR9217496).

### Statistical Analysis

Shannon and Simpson Indices were calculated from rarefied samples (80,000 reads). Both indices are common measures of diversity that reflect microbiota richness and evenness for each individual Przewalski’s horse (The Shannon index stresses the richness, whilst the Simpson index puts more weight on the evenness; Nagendra, 2002). The Good’s coverage was used to confirm the completeness of sequencing. Both diversity indices (i.e., Shannon and Simpson) and Good’s coverage were estimated using QIIME. Subsequently, both indices were tested for significant differences between study sites using One-way ANOVA (SPSS Statistics 17.0). LDA effect size (LEfSe) (Segata et al., 2011) was performed to determine differentially distributed bacterial taxa at each study site. A Kruskal–Wallis ANOVA, including all identified OTUs, was used to test whether the OTU abundance at different taxonomic levels was differentially distributed between the three study sites. OTUs violating the null hypothesis were further analyzed using a pairwise Wilcoxon test, testing whether all pairwise comparisons between subclasses within different taxonomic levels significantly agree with the taxonomic level trend. The resulting subset of vectors was used to build a LDA model from which the relative difference among taxonomic level is used to rank the OTUs. The final output thus consists of a list of OTUs that are discriminative with respect to the taxonomic level, consistent with the subclass grouping within each taxonomic level, and ranked according to the effect size with which they differentiate taxonomic levels. A size-effect threshold of 3.5 on the logarithmic LDA scale was used to identify microbiota OTUs that were specific at each study site. The LEfSe analysis was performed online, using the Galaxy workflow framework^1^. NMDS based on the Unweighted Unifrac similarities of OTU composition was applied to rank the bacterial communities using the ggplot2 in R (Version 3.5.2)^2^ (Clarke and Gorley, 2006), and a one-way ANOSIM was performed to determine differences among three study sites using the vegan package in R.

## Results

### Microbiota Composition, Richness and Diversity

**FIGURE 1:**
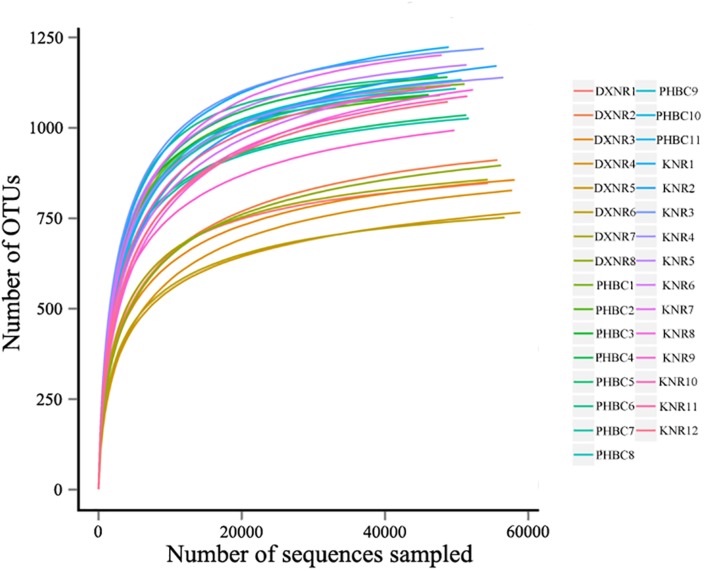
Rarefaction curves. OTU rarefaction curves of 31 fecal samples obtained from Przewalski’s horse at three study sites (reintroduced: KNR, DXNR, captive: PHBC).

**FIGURE 2:**
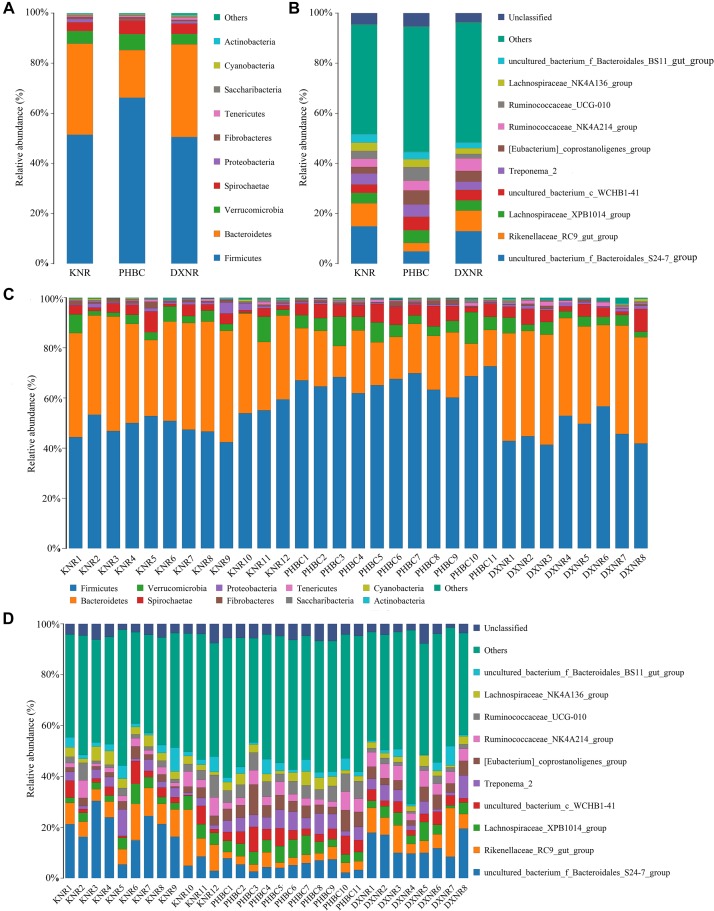
Bar chart of relative abundance. Relative abundance (%) of the ten most abundant bacteria phyla (**A** for groups, **C** for individuals) and genera (**B** for groups, **D** for individuals) obtained from 31 fecal samples of Przewalski’s horse at three study sites (reintroduced: KNR, DXNR, captive: PHBC). Others: Bacteria taxa with ≤1% abundance; Unclassified: Sequences which could not be classified.

A total of 2,088,900 effective tags were obtained from 31 fecal samples (mean ± SD: 67,384 ± 1,701). Based on a 97% sequency-similarity level, sequences were assigned to 1,392 OTUs (mean ± SD: 1,047 ± 136; for sequencing data see Supplementary Table S2). Resulting OTUs could be allocated to 17 phyla, 29 classes, 35 orders, 51 families and 146 genera. Rarefaction revealed that the number of OTUs for each sample was sufficient to carry out further analysis (Figure 1). The Good’s coverage approached 99%, suggesting that most bacteria were detected in our samples. The relative abundance of the ten most abundant phyla and genera of fecal bacteria recorded in DXNR, KNR, and PHBC is shown in Figure 2. At all study sites, Firmicutes (DXNR: 50.72%, KNR: 51.81%, PHBC: 66.29%) and Bacteroidetes (DXNR: 36.87%, KNR: 36.09%, PHBC: 18.97%) were the most prevalent phyla, followed by Verrucomicrobia (DXNR: 4.10%, KNR: 4.97%, PHBC: 6.35%) and Spirochaetae (DXNR: 4.18%, KNR: 3.42%, PHBC: 5.46%; Figure 2A). Uncultured bacteria f_Bacteroidales_S24-7 group (DXNR: 12.92%, KNR: 14.54%) and Rikenellaceae RC9 gut group were the most prevalent genera in KNR and DXNR (DXNR: 8.22%, KNR: 9.37%). In the PHBC the ten most common taxa were almost equally abundant (Figure 2B). The relative abundance of the ten most abundant phyla and genera of each individual in DXNR, KNR, and PHBC is shown in Figures 2C,D.

**FIGURE 3:**
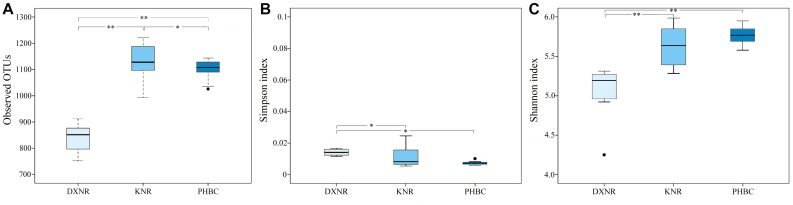
Box plot of diversity indices at three study sites (reintroduced: KNR, DXNR, captive: PHBC). **(A)** OTU richness, **(B)** Simpson diversity and **(C)** Shannon diversity. Boxes represent the interquartile range (IQR; between 25th and 75th percentiles), horizontal line inside the box defines the median, ● outliers greater than 1.5 and less than 3 times the IQR, ^*^ *p* < 0.05, ^∗∗^ *p* < 0.01.

The observed OTU richness (mean ± SD; Figure 3A) in the DXNR was significantly lower than that observed in the KNR (DXNR: 839.0 ± 56.25, KNR: 1134.0 ± 67.57; *p* < 0.01) or in the captive population at the PHBC (DXNR: 839.0 ± 56.25, PHBC: 1102.0 ± 39.40; *p* < 0.01). Moreover, OTU richness at the PHBC was significantly lower than that recorded in the KNR (PHBC: 1102.0 ± 39.40, KNR: 1134.0 ± 67.57; *p* < 0.05). The Simpson diversity (mean ± SD; Figure 3B) in the DXNR was significantly higher than that observed in KNR (DXNR: 0.022 ± 0.024, KNR: 0.011 ± 0.006; *p* < 0.05) or in the PHBC (DXNR: 0.022 ± 0.024, PHBC: 0.0073 ± 0.001; *p* < 0.05), while a negligible differences was detected between KNR and the PHBC (KNR: 0.011 ± 0.006, PHBC: 0.0073 ± 0.001; *p* > 0.05). The Shannon diversity (mean ± SD; Figure 3C) in the DXNR was significantly lower than that observed in KNR (DXNR: 5.05 ± 0.35, KNR: 5.64 ± 0.24; *p* < 0.01) or in the PHBC (DXNR: 5.05 ± 0.35, PHBC: 5.77 ± 0.12; *p* < 0.01), while a negligible differences was detected between KNR and the PHBC (KNR: 5.64 ± 0.24, PHBC: 5.77 ± 0.12; *p* > 0.05).

### Differentially Abundant Taxa Between Study Sites

**FIGURE 4:**
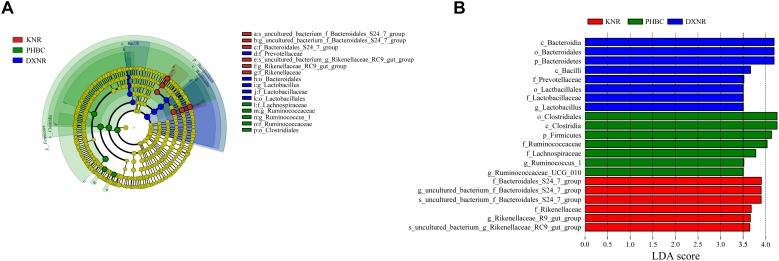
LefSe analysis. **(A)** Cladogram based on LefSe analysis showing the OTUs with significant differences between the three study sites. Taxonomic hierarchies were arranged from the inside (lower taxonomic level) to the outside (higher taxonomic level). Red, green, and blue nodes in the phylogenetic tree represent differentially abundant OTUs at the three study sites (KNR, PHBC, and DXNR, respectively). Yellow nodes represent OTUs with no significant difference. **(B)** OTUs with significant difference that have an LDA score > the threshold value of 3.5; letters in front of OTUs represent taxonomic level (*p* = phylum, c = class, o = order, *f* = family and *g* = genus).

The LEfSe analysis identified 21 OTUs that showed significantly different abundance between the three study sites (Figure 4). At phylum level, the relative abundance of Bacteroidetes was significantly higher in DXNR (0.37 ± 0.03, LDA = 4.16) compared to the other two study sites (KNR: 0.36 ± 0.08, PHBC: 0.19 ± 0.01; *p* < 0.001), while Firmicutes was significantly higher in PHBC (0.66 ± 0.01, LDA = 4.10) than at the two reintroduction sites (DXNR: 0.51 ± 0.04, KNR: 0.52 ± 0.02; *p* = 0.01). At the genus level, *Lactobacillus* in DXNR (0.03 ± 0.01, LDA = 3.56) was significantly higher than at the other two study sites (KNR: 0.003 ± 0.001, PHBC: 0.003 ± 0.001; *p* = 0.01). Relative abundance of Ruminococcus 1 (0.05 ± 0.007, LDA = 3.51) and Ruminococcaceae UCG-010 (0.05 ± 0.004, LDA = 3.52) in PHBC were significantly higher than in the two nature reserves (DXNR Ruminococcus 1: 0.01 ± 0.003, KNR Ruminococcus 1: 0.02 ± 0.003; Wilcoxon test: *p* = 0.002; DXNR Ruminococcaceae UCG-010: 0.02 ± 0.002, KNR Ruminococcaceae UCG-010: 0.03 ± 0.007; Wilcoxon test: *p* = 0.001). Relative abundance of the Uncultured bacterium f Bacteroidales S24-7 group (0.15 ± 0.03, LDA = 3.92) and the Rikenellaceae RC9 gut group (0.09 ± 0.02, LDA = 3.69) in KNR were significantly higher than the other two study sites (DXNR S24-7 group: 0.03 ± 0.02, PHBC S24-7 group: 0.05 ± 0.006; *p* = 0.002; DXNR RC9 gut group: 0.08 ± 0.02, PHBC RC9 gut group: 0.03 ± 0.003; *p* = 0.001).

### Beta Diversity

**FIGURE 5:**
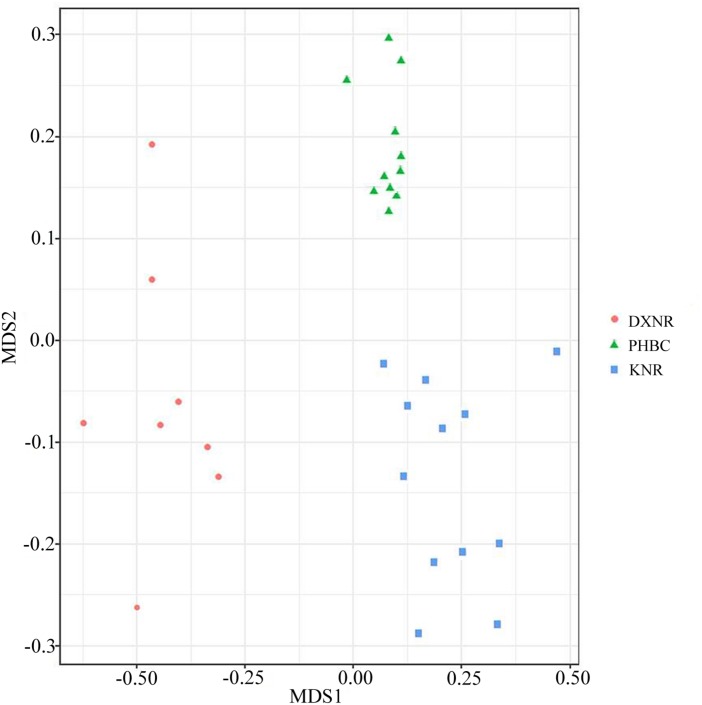
Non-metric multidimensional scaling (NMDS) analysis. NMDS scatterplot of 31 samples representing the OTU community composition of intestinal microbiota in Przewalski’s horse at three study sites (DXNR, KNR, and PHBC). The distance between points indicates the degree of difference based on Unweighted Unifrac similarities of OTU composition in each sample.

**FIGURE 6:**
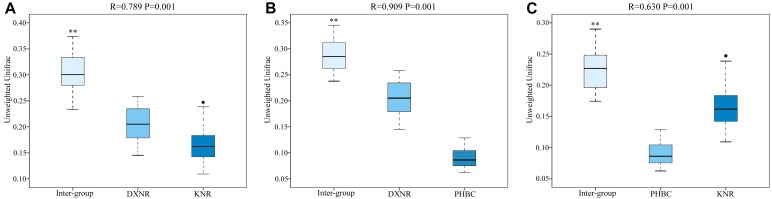
Box plot of Inter-group and Intra-group Beta distance (ANOSIM Analysis). Intra-group versus inter-group differences between DXNR and KNR **(A)**, between DXNR and PHBC **(B)** and between KNR and PHBC **(C)**. *R*-value range (0–1): *R*-values close to 0 represent no significant differences between inter-group and intra-group, *R*-values close to 1 show that inter-group differences are greater than intra-group differences. Boxes represent the interquartile range (IQR; between 25th and 75th percentiles), horizontal line inside the box defines the median, ● outliers greater than 1.5 and less than 3 times the IQR, ^∗∗^*P* < 0.01.

Operational taxonomic unit community compositions obtained from NMDS showed a similar composition within each study site but distinct compositions between study sites (Figure 5). The ANOSIM analysis revealed significant differences in bacterial communities between DXNR and KNR (*R* = 0.79, *p* = 0.001), between DXNR and PHBC (*R* = 0.91, *p* = 0.001) and between KNR and PHBC (*R* = 0.63, *p* = 0.001; Figure 6).

## Discussion

In this study, 16S-rRNA gene sequencing was used to compare the diversity and composition of intestinal microbiota of PHBC and reintroduction (DXNR and KNR). Previous studies have shown that food and nutrition are major factors affecting mammalian intestinal microbiota (Bäckhed et al., 2005; Turnbaugh et al., 2006; Schwab et al., 2011; Navarrete et al., 2012). Recent studies on hindgut fermenters have confirmed this relationship, highlighting the importance of intestinal microbiota for the health and wellbeing of horses (Costa et al., 2012; Laho et al., 2013; Elzinga et al., 2016; Metcalf et al., 2017). In our research, captive Przewalski’s horses in PHBC, were mainly fed on high protein plants and mixed feed-concentrates, which have a high nutritional value but a relatively low fiber content (Ji, 2013). By contrast, reintroduced Przewalski’s horses foraged mainly on plants with high fiber content, but with relatively low nutritional value (Meng, 2007; Chen et al., 2008; Wang et al., 2012). We therefore argue that the differences in the intestinal microbiota observed during this study were most likely linked to different feeding regimes encountered by Przewalski’s horses.

Lower Shannon diversity in DXNR compared to KNR and PHBC, while Simpson diversity showed an opposite trend compared with the Shannon index, which may be explained by the two indices weighing their components, i.e., richness and evenness, differently. This result was surprising since it stands in stark contrast to our prediction. Since previous studies have shown that intestinal microbiota diversity in wild populations is usually more diverse and complex than in captivity (Guan et al., 2017; Li et al., 2017; Metcalf et al., 2017), we expected to find this pattern also in Przewalski’s horses. Low microbiota richness at DXNR may be due to a shortage of highly nutritious food items with high protein, but low fiber content, and a limited number of preferred food plant species (Wang et al., 2012; Liu et al., 2014). The relatively simple diet was here dominated by reeds such as *P. australis*, and camelthorn, *A. sparsifolia* (Wang et al., 2012). At the PHBC, food quality was very high but variety was low, while in the KNR food plant quality and variety were relatively high (Meng, 2007; Ji, 2013). This finding corresponds to other studies (Wu et al., 2011; Yatsunenko et al., 2012), indicating that not only plant variety matters, but a combination of quality and variety determines the intestinal microbiota diversity in Przewalski’s horse. Bacterial species diversity is thus considered to represent important components of a healthy intestinal microbiome (Tuddenham and Sears, 2015). Moreover, the richness of intestinal microbiota recorded in KNR was significantly higher than that in DXNR and the PHBC, which may attributed to the variety of plant species.

LEfSe analysis showed that at the phylum level, the relative abundance of Bacteroidetes was significantly higher in DXNR than at the other two study sites, while Firmicutes was significantly higher in PHBC compared to the two reintroduction sites (Figure 4). In previous studies, researchers found that the Firmicutes to Bacteroidetes (F/B) ratio was linked to body-weight (Ley et al., 2005; Ley et al., 2006). Firmicutes can degrade complex polysaccharides into short chain fatty acids, which will result in a higher energy load than Bacteroidetes would do (Turnbaugh et al., 2006). Thus the relatively lower F/B ratio in DXNR may lead to weight loss of Przewalski’s horses. Firmicutes are the key cellulolytic bacteria in herbivores (Fernando et al., 2010). In wild ruminants, the abundance of Firmicutes in the intestine is usually higher than in captive conspecifics (Guan et al., 2017; Li et al., 2017). This is because of the relatively high fiber content of wild food plants and the higher dietary variety, compared to the relatively unbalanced diet in captivity. While ruminants are foregut fermenters and the rumen can selectively retain food particles to achieve a more thorough microbial hydrolysis, the Przewalski’s horse is a hindgut fermenter that – especially when consuming low-quality food – adopts a different strategy. This nutritional strategy is best described as “eat quickly, excrete quickly” (Duncan et al., 1990), meaning the retention time of food particles in the digestive tract is short, and therefore the degree of fiber glycolysis is low. This will result in fiber digesting bacteria to not have enough nutritional substrates and therefore not enough time to establish an effective population. Compared to reintroduced Przewalski’s horse populations, captive populations feed on high-quality forage with a relatively low fiber content. The food retention time may be longer in captive Przewalski’s horses, fiber can be well fermented and microorganisms that digest cellulose may have sufficient time to grow and reproduce.

At the genus level, the relative abundance of *Lactobacillus* is significantly higher in Przewalski’s horses from DXNR than that recorded for horses in KNR and the PHBC. Yu et al. (2018) reported that intestinal microorganism could use non-protein nitrogen to synthesize proteins. Compared to Prezwalski’s horses in the PHBC, the low quality diet in DXNR can be attributed to a lack of protein. Therefore, *Lactobacillus* may be involved in the biosynthesis of proteins to compensate for the relatively low protein content of diet in DXNR. Besides, previous studies described *Lactobacillus* to enhance the immune response of the host (Cross, 2002; Azad et al., 2018), suggesting that it may help Przewalski’s horses to withstand the relatively harsh environmental conditions in DXNR. Ruminococcus 1 and Ruminococcaceae UCG-010 abundance in the PHBC were significantly higher than those observed in DXNR and KNR. These two taxa belong to the phylum Firmicutes, which contribute to fiber digestion (Guan et al., 2017) and are thus more abundant in horses that live in captivity. Moreover, the Rikenellaceae RC9 gut group in KNR horses was significantly higher than that recorded in horses roaming the DXNR and kept in the PHBC. Although this group was recently identified in a number of other large herbivores, including elephants, horses, sheep and cattle (Ilmberger et al., 2014; Li et al., 2014; Rodriguez et al., 2015; Huang et al., 2018), the function of these microorganisms in the gut of herbivores remains unresolved. At the order level, the relative abundance of Clostridiales is significantly higher in Przewalski’s horses from PHBC than horses in KNR and DXNR, there is a positive correlation between Clostridiales and dietary protein content and protein digestibility (Bermingham et al., 2013), which can be explained by the high protein diet in captivity.

Non-metric multi-dimensional scaling and ANOSIM clearly indicated a distinct separation between the three study sites (Figures 5, 6), suggesting that the composition of bacterial communities was significantly different between DXNR, KNR, and PHBC. Although diet plays the most important role in altering the composition and structure of intestinal microbiota communities, several other factors may also play an important role. These include the age of the host, its body condition, or environmental factors such as climate or soil composition (Hill et al., 2005; Jandhyala et al., 2015). In order to keep the impact of those factors at a minimum, we standardized our sampling efforts by including only individuals of similar age and by collecting all fecal samples in the same months under similar weather conditions (i.e., in July). Overall, the core microbiota of Przewalski’s horses at the three study sites were very similar (Figure 2). Firmicutes and Bacteroidetes represented the most abundant phyla, which are known to constitute the bacterial community of many mammalian species such as reindeer (*Rangifer tarandus*), Asian buffalo (*Bubalus arnee*), and musk deer (*Moschus* spp.) (Sundset et al., 2007; Pandya et al., 2010; Hu et al., 2017; Li et al., 2017). This general pattern in intestinal microbiota composition indicates that these phyla play an important ecological and functional role in the intestine of mammals and that this symbiotic relationship has developed relatively early during mammalian evolution (Shanks et al., 2011).

Intestinal microbial communities should be considered as vital factors that provide insights into a species’ nutrition and digestion. Our study highlights the importance of diet in shaping the intestinal microbiota of Przewalski’s horses under three different feeding regimes and clearly indicates that reintroduced individuals need to adapt to the higher fiber and roughage content of food plants in the wild. This was particularly true for the diet consumed in DXNR, which led to a high relative abundance of the phylum Bacteriodetes. These taxa suggest that food quality was relatively poor at DXNR with a high fiber content and low nutritious value. Moreover, the relatively high abundance of the genus *Lactobacillus* may help Przewalski’s horses to withstand the harsh environmental conditions in DXNR. We therefore propose that microbiota identified from Przewalski’s horses at existing reintroduction sites – combined with a detailed knowledge of consumed and available food plants at those sites (e.g., Meng, 2007; Wang et al., 2012 in our study) – could guide the selection of future reintroduction sites for Przewalski’s horses.

## Ethics Statement

This study was carried out in accordance with the recommendations of the Institute of Animal Care and the Ethics Committee of Beijing Forestry University. The Ethics Committee of Beijing Forestry University also approved the protocol. The management authority of Kalamaili Nature Reserve, Dunhuang Xihu Nature Reserve and the Xinjiang Przewalski Horse Breeding Center approved the collection of Przewalski horse fecal samples.

## Author Contributions

YML and DH conceived and designed the experiments. YL and KZ carried out the DNA extraction and analyzed the data. YL and KL participated in sample collection. YML and TW wrote the manuscript. All authors read and approved the final manuscript.

## Conflict of Interest Statement

The authors declare that the research was conducted in the absence of any commercial or financial relationships that could be construed as a potential conflict of interest.

## Footnotes

1 http://huttenhower.sph.harvard.edu/galaxy/

2 http://www.r-project.org
